# Supplementary material for: Antidepressant use and all-cause mortality in depressed individuals: A real-world cohort study
Source: PLoS One. 2025 Jul 11;20(7):e0327844. doi: 10.1371/journal.pone.0327844 (PMC12250549; doi:10.1371/journal.pone.0327844)
Supplement: S1 File — S1 Table. Characteristics of Patients (based on PHQ-9 ≥ 10) Receiving or Not Receiving Antidepressants, before and after PSM. S2 Table. Associations between Antidepressant Use and Death in the Crude Analysis, Multivariable Analysis, and Propensity-Score Analyses. (DOCX) [file pone.0327844.s001.docx]

Antidepressant Use and All-Cause Mortality in Depressed Individuals: A Real-World Cohort Study

Supplementary Material

Table S1. Characteristics of Patients (based on PHQ-9≥10) Receiving or Not Receiving Antidepressants, before and after PSM.

|  | Unmatched Patients | | P | Propensity-Score–Matched Patients | | P |
| --- | --- | --- | --- | --- | --- | --- |
|  | No Antidepressants | Antidepressants | value | No Antidepressants | Antidepressants | value |
| N | 2022 | 944 |  | 891 | 891 |  |
| Age, years | 47.38 ± 16.83 | 52.50 ± 14.24 | <0.001 | 51.63 ± 16.17 | 51.85 ± 14.20 | 0.765 |
| Gender, n (%) | 801 (39.6) | 279 (29.6) | <0.001 | 286 (32.6) | 275 (31.3) | 0.609 |
| Race, n (%) |  |  | <0.001 |  |  | <0.001 |
| Non-Hispanic White | 726 (35.9) | 524 (55.5) |  | 430 (49) | 467 (53.2) |  |
| Non-Hispanic Black | 502 (24.8) | 162 (17.2) |  | 223 (25.4) | 154 (17.5) |  |
| Others | 794 (39.3) | 258 (27.3) |  | 225 (25.6) | 257 (29.3) |  |
| Education level, n (%) |  |  | <0.001 |  |  | 0.817 |
| Less than high school | 792 (39.2) | 267 (28.3) |  | 262 (29.8) | 261 (29.7) |  |
| High school or equivalent | 480 (23.7) | 240 (25.4) |  | 213 (24.3) | 224 (25.5) |  |
| College or above | 750 (37.1) | 437 (46.3) |  | 403 (45.9) | 393 (44.8) |  |
| Marital status, n (%) |  |  | <0.001 |  |  | 0.954 |
| never_married | 459 (22.7) | 154 (16.3) |  | 145 (16.5) | 148 (16.9) |  |
| Married/cohabiting | 635 (31.4) | 370 (39.2) |  | 346 (39.4) | 340 (38.7) |  |
| Others | 928 (45.9) | 420 (44.5) |  | 387 (44.1) | 390 (44.4) |  |
| PIR, n (%) |  |  | 0.017 |  |  | 0.393 |
| ≤1.3 | 998 (49.4) | 429 (45.4) |  | 400 (45.6) | 401 (45.7) |  |
| 1.3–3.5 | 607 (30) | 296 (31.4) |  | 300 (34.2) | 274 (31.2) |  |
| >3.5 | 226 (11.2) | 140 (14.8) |  | 115 (13.1) | 127 (14.5) |  |
| Unknown | 191 (9.4) | 79 (8.4) |  | 63 (7.2) | 76 (8.7) |  |
| Drinking, n (%) |  |  | 0.022 |  |  | 0.126 |
| No | 499 (24.7) | 234 (24.8) |  | 190 (21.6) | 219 (24.9) |  |
| Yes | 1253 (62) | 549 (58.2) |  | 553 (63) | 512 (58.3) |  |
| Unkown | 270 (13.4) | 161 (17.1) |  | 135 (15.4) | 147 (16.7) |  |
| Smoking, n (%) |  |  | 0.002 |  |  | 0.843 |
| Never | 850 (42) | 348 (36.9) |  | 323 (36.8) | 326 (37.1) |  |
| Former | 415 (20.5) | 245 (26) |  | 212 (24.1) | 220 (25.1) |  |
| Now | 757 (37.4) | 351 (37.2) |  | 343 (39.1) | 332 (37.8) |  |
| Physical activity, n (%) | 572 (28.3) | 221 (23.4) | 0.006 | 218 (24.8) | 205 (23.3) | 0.503 |
| BMI, n (%) |  |  | <0.001 |  |  | 0.105 |
| Normal/Underweight | 548 (27.1) | 168 (17.8) |  | 167 (19) | 168 (19.1) |  |
| Overweight | 568 (28.1) | 207 (21.9) |  | 232 (26.4) | 195 (22.2) |  |
| Type 2 diabetes, n (%) | 450 (22.3) | 281 (29.8) | <0.001 | 235 (26.8) | 251 (28.6) | 0.424 |
| CVD, n (%) | 339 (16.8) | 241 (25.5) | <0.001 | 209 (23.8) | 214 (24.4) | 0.823 |
| Hypertensive, n (%) | 1159 (57.3) | 392 (41.5) | <0.001 | 402 (45.8) | 378 (43.1) | 0.269 |
| Arthritis, n (%) | 768 (38) | 565 (59.9) | <0.001 | 482 (54.9) | 499 (56.8) | 0.442 |
| CKD, n (%) | 358 (17.7) | 182 (19.3) | 0.325 | 168 (19.1) | 164 (18.7) | 0.855 |
| Cancer, n (%) | 201 (9.9) | 133 (14.1) | 0.001 | 116 (13.2) | 117 (13.3) | 1.000 |
| Death, n (%) | 247 (12.2%) | 128 (13.6%) | 0.305 | 130 (14.8) | 114 (13.0) | 0.301 |
| Total cholesterol, mg/dL | 194.57 ± 44.47 | 196.86 ± 44.61 | 0.307 | 196.57 ± 46.11 | 197.05 ± 44.85 | 0.828 |
| Triglyceride, mg/dL | 143.14 ± 144.58 | 147.69 ± 110.41 | 0.021 | 148.13 ± 164.40 | 148.25 ± 112.42 | 0.991 |

N represents the number of patients in each group. PHQ-9: Patient Health Questionnaire-9. PSM: Propensity-Score Matching. PIR: Poverty Income Ratio. CVD: Cardiovascular Disease. CKD: Chronic Kidney Disease. Depression was determined by the 9-item Patient Health Questionnaire (PHQ-9) score of ≥10.

Table S2. Associations between Antidepressant Use and Death in the Crude Analysis, Multivariable Analysis, and Propensity-Score Analyses.

| Analysis | Death |
| --- | --- |
| No. of events/no. of patients at risk (%) |  |
| Antidepressant Users | 128/944 (13.6%) |
| Non-Antidepressant Users | 247/2022 (12.2%) |
| Crude analysis — hazard ratio (95% CI) P value | 1.26 (1.02, 1.56) 0.0322 |
| Adjust for all covariates^*^ — hazard ratio (95% CI) P value | 1.01 (0.80, 1.27) 0.9293 |
| Adjust for PS^*^ — hazard ratio (95% CI) P value | 1.01 (0.80, 1.27) 0.9407 |
| Estimate of treatment effect using IPTW — hazard ratio (95% CI) P value^†^ |  |
| ATT | 0.98 (0.78, 1.24) 0.8894 |
| ATC | 1.12 (0.85, 1.46) 0.4192 |
| ATE | 1.07 (0.84, 1.36) 0.5785 |
| Estimate of treatment effect using PS match—hazard ratio (95% CI) P value^†^ |  |
| ATT | 1.12 (0.86, 1.45) 0.3960 |
| ATC | 1.46 (0.71, 2.99) 0.3004 |
| ATE | 1.31 (0.75, 2.29) 0.3351 |

PS: propensity score, ATT: average treatment effect for treated, ATC: average treatment effect for control, ATE: average treatment effect for all.
^*^Shown is the hazard ratio from the weighted multivariable Cox proportional-hazards model, with adjustment for age, gender, race, education level, marital status, and the poverty-income ratio, alcohol consumption, smoking status, physical activity, Body mass index, diabetes, cardiovascular disease, hypertension, arthritis, chronic kidney disease, and cancer. The analysis included all 5947 patients. The propensity score is estimated by incorporating all the aforementioned covariates.
^†^Shown is the analysis with hazard ratio from the multivariable Cox proportional-hazards model in the matched data with matching the propensity score or inverse probability of treatment weighting using the propensity score.

Depression was determined by the 9-item Patient Health Questionnaire (PHQ-9) score of ≥10.
