# Supplementary material for: Antidepressant use and all-cause mortality in depressed individuals: A real-world cohort study
Source: PLoS One. 2025 Jul 11;20(7):e0327844. doi: 10.1371/journal.pone.0327844 (PMC12250549; doi:10.1371/journal.pone.0327844)
Supplement: Attachment — Submitted filename: Comments1.doc [file pone.0327844.s002.doc]

To me it is an lInteresting topic, but I have some comments on the discussion part and others especially on the reference, some parts the reference method is vancouver, and some parts Harvard. As to me it is possible to use one reference style.

1. Does all types antidepressants are safe especially TCAs

2. What are the challenges and Limitations of the study

3. Recommendations of the study

4. Antidepressant Use and All-Cause Mortality in Depressed Individuals, what is ur findings on the mortality in clients with antidepressants?

5. Antidepressant users (n=3,925) had a crude mortality rate of 16.5%, compared to12.2% in non-users (n=2,022). Is proportional ? control i.e non users and antidepressants users. If yes , this results shows that mortality is higher in this users of antidepressants.
