# Supplementary material for: Antidepressant use and all-cause mortality in depressed individuals: A real-world cohort study
Source: PLoS One. 2025 Jul 11;20(7):e0327844. doi: 10.1371/journal.pone.0327844 (PMC12250549; doi:10.1371/journal.pone.0327844)
Supplement: Attachment — Submitted filename: Response to Reviewers.docx [file pone.0327844.s004.docx]

Manuscript ID: PONE-D-25-19212

Title: Antidepressant use and all-cause mortality in depressed individuals: a real-world cohort study’

Dear Dr. Kuo-Cherh Huang and Reviewers,

We sincerely appreciate the time and effort you have dedicated to reviewing our manuscript and providing constructive feedback. We are grateful for the opportunity to revise our work and address the reviewers' comments. In order to facilitate your review, all changes and additions made in the revised manuscript are highlighted in blue. Below, we provide a point-by-point response to each comment, detailing the changes made to the manuscript.

Journal Requirements

1. PLOS ONE Style and File Naming

**Response:** We have revised our manuscript according to the PLOS ONE style templates, including formatting and file naming requirements. All references have been checked and uniformly formatted in Vancouver style to ensure completeness and accuracy. We have carefully revised Table 1 according to the journal's formatting requirements. To improve space efficiency and maintain clarity, we have consolidated the originally split table into a single presentation. Additionally, we have modified the presentation of binary categorical variables (e.g., Gender) by displaying only one category with its percentage (Male, %), while maintaining all statistical values. This adjustment preserves the complete information while optimizing the table's layout to fit within one page. All data remain unchanged from the original submission. Table S1 (Supplementary Material) has been updated following the same revisions.

2. Data Anonymization

**Response:** Our study uses the publicly available NHANES database, which does not contain any personally identifiable information. No personal data is included in our submission.

3. Supporting Information Captions

**Response:** We have now included the captions for all Supporting Information files at the end of the manuscript. All in-text citations to Supporting Information remain accurate and unchanged..

4. Reference List Review

**Response:** We have carefully reviewed and revised all references to ensure completeness and accuracy, and standardized them to Vancouver style. In response to reviewer concerns regarding tricyclic antidepressants (TCAs), we have added references 26 and 27. The placement of these additions does not alter the sequence of the other references since these two citations come after all the other references.

**Reviewers' Comments**

Reviewer #1: This is an intelligent review of an important issue.

I only question on sentence in the discussion section:

"Taken together, these results demonstrate no statistically meaningful link between depression and mortality risk, irrespective of whether depression was classified by PHQ-9 scores alone or in combination with antidepressant use."

The study was not designed to study depression and mortality.

The sentence could be phrased more clearly, or deleted entirely.

**Response:** Thank you very much for your positive comments and for highlighting this important point. We agree with your observation that the sentence in question does not accurately reflect the study design and could be misleading. In accordance with your suggestion, we have deleted the sentence from the discussion section. We believe this change improves the clarity and focus of our manuscript.

Thank you again for your constructive comment, which has helped improve our paper.

Reviewer #2: To me it is an lInteresting topic, but I have some comments on the discussion part and others especially on the reference, some parts the reference method is vancouver, and some parts Harvard. As to me it is possible to use one reference style.

**Response:** We sincerely appreciate your diligent review and valuable feedback on our manuscript. In response to your concerns regarding reference formatting, we have carefully reviewed all citations and standardized them according to the Vancouver style to ensure consistency. Additionally, we have revalidated and corrected the accuracy of all references.

**For example:**

**Original:**

1. World Health Organization. (2023). Depressive disorder (depression). https://www.who.int/news-room/fact-sheets/detail/e-coli [Accessed January 27 2025].

**Revised:**

1. World Health Organization [Internet]. Depressive disorder (depression). 2023 [cited 2025 Jan 27]. Available from: https://www.who.int/news-room/fact-sheets/detail/depression

**Original:**

17. Rovin BH, Adler SG, Barratt J, Bridoux F, Burdge KA, Chan TM, et al. KDIGO 2021 Clinical Practice Guideline for the Management of Glomerular Diseases. Kidney International. (2021) 100:S1-S276. doi:10.1016/j.kint.2021.05.021

**Revised:**

17. Rovin BH, Adler SG, Barratt J, Bridoux F, Burdge KA, Chan TM, et al. KDIGO 2021 clinical practice guideline for the management of glomerular diseases. Kidney Int. 2021;100:S1-S276. doi:10.1016/j.kint.2021.05.021

We have carefully checked and revised all other references line by line to ensure they conform to the Vancouver style. Thank you again for your helpful suggestions.

1. Does all types antidepressants are safe especially TCAs

**Response:** We sincerely appreciate your thoughtful question regarding the safety of different antidepressant types, particularly TCAs. Your comment highlights an important limitation in our study that deserves further clarification. We understand your concern stems from the fact that different classes of antidepressants have distinct pharmacological profiles and safety considerations. TCAs, in particular, have been associated with greater cardiovascular risks compared to some other antidepressants. Our broad conclusion about antidepressant safety without class-specific analysis could potentially mask important differences in safety profiles.

In our study, we analyzed antidepressants as a collective group due to several methodological constraints. First, many participants were on multiple antidepressants or switched medications during the follow-up period, making class-specific analysis challenging. Second, the NHANES database, while comprehensive, does not provide sufficient granularity on medication dosage, duration, and adherence to conduct robust class-specific analyses. Additionally, stratifying by antidepressant class would have significantly reduced statistical power for each subgroup.

We acknowledge this important limitation and have revised our manuscript to more clearly articulate the scope of our findings. We have modified our conclusion to emphasize that our results reflect the overall safety profile of antidepressants as a collective group, while acknowledging that safety profiles may vary among specific classes.

**In the manuscript, we have made the following revisions:**

**Discussion section:**

**Add a paragraph (paragraph 3, around line 259-266):** "It should be emphasized that our findings reflect the average effect of antidepressant use as a whole and should not be construed as evidence that all classes of antidepressants share identical safety profiles. In this study, antidepressants were analyzed collectively, without differentiation by specific class. However, various classes—such as selective serotonin reuptake inhibitors (SSRIs), serotonin-norepinephrine reuptake inhibitors (SNRIs), tricyclic antidepressants (TCAs), and monoamine oxidase inhibitors (MAOIs)—possess distinct pharmacological characteristics and may present differing safety concerns. For example, TCAs have been associated with a higher risk of cardiovascular adverse events compared to some other antidepressants[26,27]."

**Limitations section (around line 310-313):**

**Original:** "Furthermore, the study did not differentiate between different types of antidepressants, limiting our ability to assess the safety of specific antidepressant medications."

**Expand:** "Fifth, the study did not differentiate between different types of antidepressants, limiting our ability to assess the safety of specific antidepressant classes. This is particularly important as different antidepressants—such as SSRIs, SNRIs, TCAs, and MAOIs—have distinct pharmacological profiles and potentially different safety considerations."

2. What are the challenges and Limitations of the study

**Response:** We sincerely appreciate your question regarding the challenges and limitations of our study. This is indeed a critical aspect of scientific research that warrants thorough discussion.

We understand that a comprehensive acknowledgment of study limitations is essential for the proper interpretation of research findings. In our manuscript, we have included a limitations section, but we acknowledge that a more detailed discussion would strengthen the paper and provide readers with a clearer understanding of the constraints under which our conclusions should be interpreted.

In our study, we faced several methodological challenges. First, while the NHANES database provides a nationally representative sample with standardized data collection protocols, it has inherent limitations in terms of medication details, such as dosage, duration, and adherence patterns. Second, despite our rigorous statistical approaches to control for confounding, including propensity score matching and inverse probability of treatment weighting, residual confounding from unmeasured variables remains a possibility. Third, the operational definition of depression using PHQ-9 scores or antidepressant use, while pragmatic, may not capture the full spectrum of depressive disorders with the same precision as structured clinical interviews.

We have revised our manuscript to provide a more comprehensive discussion of these limitations and their potential impact on our findings. The expanded limitations section now addresses additional challenges such as the inability to account for changes in depression severity over time, potential selection bias, and the generalizability of our findings to populations outside the United States.

**In the manuscript, we have made the following revisions:**

**Discussion section (around line 266-272):**

**Add a paragraph acknowledging key methodological challenges:** "Additionally, our study faced several methodological challenges worth noting. The assessment of depression using PHQ-9 or antidepressant use, while practical for large epidemiological studies, may not capture the nuanced clinical presentation of depression. Moreover, the NHANES database lacks detailed information on antidepressant dosage, duration, and adherence patterns, which may influence mortality outcomes. These limitations may hinder causal inference, despite the application of robust statistical methods to control for confounders."

**Limitations section (around line 299-321):**

**Original:** "Although this study has several strengths, it also has certain limitations. The study's findings are limited to adults aged 20 and older, and may not be applicable to adolescents. Second, although we employed multiple statistical methods to control for confounding factors, there may still be unmeasurable or unknown confounders affecting the results, such as treatment adherence, changes in depression severity, and other undocumented concurrent medications. Furthermore, the study did not differentiate between different types of antidepressants, limiting our ability to assess the safety of specific antidepressant medications. Finally, while NHANES provides a nationally representative sample, extrapolating these results to other countries or regions requires caution, as genetic backgrounds, lifestyle factors, and medical practices may differ across populations."

**Revised:** "Despite its strengths, our study has several important limitations that warrant consideration. First, our findings are limited to adults aged 20 and older and may not be applicable to adolescents or young adults under 20, a population with unique considerations regarding antidepressant use and safety. Second, although we employed multiple statistical methods to control for confounding factors, residual confounding from unmeasured variables remains possible. Specifically, we lacked detailed information on treatment adherence, changes in depression severity over time, concurrent psychotherapy, and undocumented medications that might interact with antidepressants. Third, the NHANES database, while comprehensive, provides limited information on medication dosage, duration, and patterns of use, which may influence mortality outcomes. Fourth, our operational definition of depression using PHQ-9 scores or antidepressant use, while pragmatic for large-scale epidemiological studies, may not capture the full clinical spectrum of depressive disorders with the same precision as structured clinical interviews. Fifth, the study did not differentiate between different types of antidepressants, limiting our ability to assess the safety of specific antidepressant classes. This is particularly important as different antidepressants—such as SSRIs, SNRIs, TCAs, and MAOIs—have distinct pharmacological profiles and potentially different safety considerations. Sixth, we could not account for potential selection bias in antidepressant prescription, where physicians might preferentially prescribe certain antidepressants to patients with specific health profiles. Finally, while NHANES provides a nationally representative sample of the US population, extrapolating these results to other countries or regions requires caution, as genetic backgrounds, healthcare systems, prescribing practices, and lifestyle factors may differ substantially across populations. These limitations highlight the need for further research with more detailed medication data and longer follow-up periods to understand better the long-term safety of specific antidepressant classes in various subpopulations."

3. Recommendations of the study

**Response:** Thank you for your valuable suggestion regarding the recommendations of our study. We appreciate the opportunity to elaborate on the practical implications of our findings.

We understand that translating research findings into actionable recommendations is crucial for enhancing the clinical utility and impact of scientific research. While our manuscript briefly discusses some clinical implications, we acknowledge that a more comprehensive and structured presentation of recommendations would strengthen the paper.

Based on our findings that antidepressant use does not significantly impact all-cause mortality in individuals with depression, we have expanded our recommendations for clinical practice and healthcare policy.

We have revised our manuscript to include a dedicated "Recommendations" subsection within the Discussion section, providing more specific and actionable guidance based on our findings. As you rightly noted, while our pooled analysis found no overall mortality risk with antidepressants, certain classes (e.g., TCAs) may carry distinct risks. The original recommendation to prioritize efficacy without mortality concerns might oversimplify clinical decision-making, especially for high-risk patients or specific drug types.

**In the manuscript, we have made the following revisions:**

**Discussion section (around line 276-283):**

**Original:** "For clinical practice, we recommend that physicians focus more on the therapeutic effects of antidepressants rather than being overly concerned about their impact on mortality risk. However, given the heterogeneity of depressed patients, more research is needed for specific subgroups (such as elderly patients and those with multiple comorbidities) to optimize individualized treatment strategies."

**Revised:** "For clinical practice, while antidepressants as a collective group show no significant mortality risk, clinicians should still consider individual patient factors (e.g., comorbidities, age) and drug-specific safety profiles when prescribing. Pre-treatment safety assessments remain essential, particularly for agents with known risks (e.g., TCAs in cardiovascular disease). In healthcare policy, guidelines for depression management should recognize the overall safety of antidepressants in relation to mortality outcomes, while also emphasizing the significance of appropriate prescribing practices. Mental health literacy programs should address misconceptions about the safety of antidepressants to diminish stigma and overcome barriers to treatment."

4. Antidepressant Use and All-Cause Mortality in Depressed Individuals, what is ur findings on the mortality in clients with antidepressants?

**Response:** Thank you for your question regarding our findings on mortality in clients with antidepressants. We appreciate the opportunity to clarify these important results. Our comprehensive analyses yielded several key findings:

In our nationally representative cohort of 5,947 depressed adults (median follow-up 82 months), we observed:

• Crude mortality rates of 16.5% (647/3,925) in antidepressant users vs. 12.2% (247/2,022) in non-users

• Initial unadjusted analysis showed a non-significant trend (HR=1.18, 95% CI 0.95-1.47, P=0.126)

• Full covariate adjustment attenuated this association (adjusted HR=0.92, 95% CI 0.75-1.13, P=0.435)

To address potential confounding, we employed multiple rigorous methods:

• Propensity score weighting (IPTW): HR=0.96 (0.80-1.16, P=0.707)

• Propensity score matching: HR=0.83 (0.65-1.06, P=0.137)

• Sensitivity analyses using PHQ-9≥10 definition confirmed robustness

The complete attenuation of effect after adjustment suggests the initial difference likely reflected confounding by indication rather than medication effects. Our comprehensive analyses consistently demonstrated no statistically significant association between overall antidepressant use and mortality risk.

**We have revised our manuscript to more clearly articulate these findings in the Discussion sections, emphasizing both the initial crude mortality rates and the adjusted analyses that account for potential confounding factors.**

**Discussion section (around line 223-237):**

**Original:** "This study investigated the link between the utilization of antidepressants and all-cause mortality among individuals with depression. This study analyzed 5,947 participants with depression from a nationally representative NHANES cohort, tracked over a median of 82 months. This study utilized two different criteria to define depression in its analyses. Depression was operationalized as a PHQ-9 score of 10 or higher or current use of antidepressants. Initial unadjusted analyses revealed a slightly increased risk of death among individuals using antidepressants compared to those not using them, though this difference was not statistically significant (HR=1.18, 95% CI 0.95-1.47, p=0.126). Following adjustment for relevant covariates, the risk of death among antidepressant users slightly decreased, remaining statistically insignificant (HR=0.92, 95% CI 0.75-1.13, p=0.435). Further analyses consistently showed no statistically significant difference in mortality risk between antidepressant users and non-users. Using IPTW as the primary analytical method, the average treatment effect on the treated (ATT) HR was 0.96 (95% CI: 0.80–1.16, P = 0.707). Sensitivity analyses, which defined depression strictly based on depressive symptoms (PHQ-9 ≥ 10), produced similar findings. In the IPTW model, the ATT was not significant (HR = 0.98, 95% CI 0.78–1.24, P = 0.889; refer to Supplementary Tables S2).The robustness of these findings across different analytical approaches reinforces the conclusion that antidepressant treatment does not significantly influence mortality outcomes in individuals with depression."

**Revised:** "Our study found no statistically significant association between the overall use of antidepressants and all-cause mortality after comprehensive adjustment for potential confounding factors. While the unadjusted analysis showed a slightly higher crude mortality rate among antidepressant users (16.5% vs. 12.2%), this difference was not statistically significant (HR=1.18, 95% CI 0.95-1.47, P=0.126). After adjusting for relevant covariates, the hazard ratio decreased to 0.92 (95% CI 0.75-1.13, P=0.435), suggesting that the initial trend may have been influenced by differences in baseline characteristics between the groups. Our propensity score analyses further confirmed the absence of a significant association between overall antidepressant use and all-cause mortality risk. Sensitivity analyses, which defined depression strictly based on depressive symptoms (PHQ-9 ≥ 10), produced similar findings. These findings underscore an important methodological consideration in real-world research: the apparent differences in crude mortality rates between groups may be largely explained by confounding factors. Our results highlight the necessity of appropriate statistical adjustment, especially when baseline characteristics differ substantially between comparison groups. Collectively, our study provides important evidence regarding the overall safety of antidepressants with respect to mortality outcomes in individuals with depression."

5. Antidepressant users (n=3,925) had a crude mortality rate of 16.5%, compared to12.2% in non-users (n=2,022). Is proportional ? control i.e non users and antidepressants users. If yes , this results shows that mortality is higher in this users of antidepressants.

**Response:** Thank you for your astute inquiry regarding the proportionality of our study groups and the interpretation of the crude mortality rates. You have raised an important methodological point that warrants clarification.

You are correct in noting that our study included 3,925 antidepressant users and 2,022 non-users, creating an approximately 2:1 ratio between the groups. You also correctly observed that the crude mortality rate was higher in antidepressant users (16.5%) compared to non-users (12.2%). This imbalance in both group sizes and crude mortality rates deserves careful consideration.

The group size ratio of approximately 2:1 mirrors the real distribution of antidepressant utilization among depressed patients in the NHANES dataset, which was not deliberately regulated by us and does not inherently invalidate the results. The higher crude mortality rate in antidepressant users can be largely attributed to significant baseline differences between the groups, including older age (55.3±16.0 vs. 47.4±16.8 years, P<0.001) and higher prevalence of chronic conditions in the antidepressant group. Modern statistical methods can account for such imbalances, which is why we employed multiple analytical approaches, including propensity score matching and inverse probability of treatment weighting.

**Based on your question, we have made the following revisions:**

**Methods section (around line 136):**

Enhance the description of statistical methods to address group imbalance:

**Original:** "To further account for confounding, we employed propensity score methods."

**Revised:** "To further account for the imbalance in group sizes and confounding, we employed propensity score methods."

**Results section (around line 181-189):**

**Original:** "Among the 5,947 individuals, there were 894 deaths(15.0%). Antidepressant users accounted for 3,925 individuals, of whom 647 died(16.5%).In contrast, non-antidepressant users comprised 2,022 individuals, with 247 deaths(12.2%). "

**Revised:** "Among the 5,947 individuals with depression, 894 deaths (15.0%) occurred during the median follow-up period of 82 months. Although the crude mortality rate appeared higher in antidepressant users (647/3,925, 16.5%) compared to non-users (247/2,022, 12.2%), this imbalance reflects the real-world distribution of antidepressant use among individuals with depression in the NHANES dataset. The higher crude mortality rate in antidepressant users can be largely attributed to significant baseline differences between the groups, including older age (55.3±16.0 vs. 47.4±16.8 years, P<0.001) and higher prevalence of chronic conditions in the antidepressant group. To address this potential confounding, we employed multiple statistical approaches including multivariable adjustment, propensity score matching, and IPTW. "

**Discussion section (around line 232-237):**

**Add a paragraph:** "These findings underscore an important methodological consideration in real-world research: the apparent differences in crude mortality rates between groups may be largely explained by confounding factors. Our results highlight the necessity of appropriate statistical adjustment, especially when baseline characteristics differ substantially between comparison groups. Collectively, our study provides important evidence regarding the overall safety of antidepressants with respect to mortality outcomes in individuals with depression."

**Additionally, we have also revised the conclusion section.**

**Abstract (Conclusion section):**

**Original:** "Antidepressant use in individuals with depression does not significantly impact all-cause mortality, indicating their safety concerning mortality risk."

**Revised:** "All-cause mortality is not significantly affected by the overall use of antidepressants in individuals with depression; however, future studies should investigate safety differences between specific drug classes."

**Conclusion section(around line 323-329):**

**Original:** "Analysis of this population-based cohort revealed no statistically meaningful link between antidepressant treatment and mortality risk among depressed participants. While the results offer reassurance about the safety of antidepressants in terms of mortality, further research is needed to explore potential benefits or risks in specific subpopulations."

**Revised:** "Analysis of this population-based cohort revealed no statistically meaningful link between antidepressant treatment, analyzed as a collective group, and mortality risk among depressed participants. While these results offer general reassurance about antidepressant safety regarding mortality outcomes, the limitations of our study, including the inability to differentiate between antidepressant classes and potential residual confounding, should be considered when interpreting these findings. Further research is needed to address these limitations and explore the potential benefits or risks of different antidepressants for specific subpopulations."

We hope that these revisions satisfactorily address all concerns raised by the editor and reviewers. Thank you again for your valuable feedback and consideration.

Sincerely,
Shaoyu Zhou
On behalf of all authors

Reference

26.	Taylor D, Poulou S, Clark I. The cardiovascular safety of tricyclic antidepressants in overdose and in clinical use. Ther Adv Psychopharmacol. 2024;14:20451253241243297. doi:10.1177/20451253241243297

27.	Jang HY, Kim JH, Song YK, Shin JY, Lee HY, Ahn YM, et al. Antidepressant use and the risk of major adverse cardiovascular events in patients without known cardiovascular disease: a retrospective cohort study. Front Pharmacol. 2020;11:594474. doi:10.3389/fphar.2020.594474
